# Using forensic analytics and machine learning to detect bribe payments in regime-switching environments: Evidence from the India demonetization

**DOI:** 10.1371/journal.pone.0268965

**Published:** 2022-06-09

**Authors:** Ben Charoenwong, Pooja Reddy

**Affiliations:** 1 National University of Singapore Business School, Singapore, Singapore; 2 India Institute of Technology Kharagpur, Kharagpur, India; Universiti Teknologi Malaysia - Main Campus Skudai: Universiti Teknologi Malaysia, MALAYSIA

## Abstract

We use a rich set of transaction data from a large retailer in India and a dataset on bribe payments to train random forest and XGBoost models using empirical measures guided by Benford’s Law, a commonly used tool in forensic analytics. We evaluate the performance around the 2016 Indian Demonetization, which affects the distribution of legal tender notes in India, and find that models using only pre-2016 data or post-2016 data for both training and testing data had F1 score ranges around 90%, suggesting that these models and Benford’s law criteria contain meaningful information for detecting bribe payments. However, the performance for models trained in one regime and tested in another falls dramatically to less than 10%, highlighting the role of the institutional setting when using financial data analytics in an environment subject to regime shifts.

## 1. Introduction

Forensic analytics has been used extensively in various applications to detect irregularities in financial statements [1] and illicit financial flows [2]. Many of the applications compare how distributions of values line up with Benford’s Law, which shows how the leading digits of values line up from 0 to 9 relative to what a naturally occurring set of values. Whether deviations from Benford’s Law can be used in detecting suspicious transactions depends on the means of payment. For example, cash transactions for illicit activity may cluster around legal tender values, like multiples of $100 if using the United States dollar. However, recent advances in payment technology, particularly in the digital payments space such as cryptocurrency (which leaves a publicly observable trail of transactions) and wallet-to-wallet transfers may affect how illicit transactions are distributed through the lens of Benford’s Law. To our knowledge, no paper has studied the impact of such “regime shifts” on the performance of forensic analytic models.

In this paper, we study whether Benford’s Law is a viable methodology to detect bribe payments, and whether it depends on the economic regimes, by using a setting from India straddling the 2016 Demonetization event which changes the paper currency notes that are legal tender. We consider the use of forensic analysis in combination with simple machine learning models to detect bribe payments in a dataset containing identified bribe and non-bribe payments. We hypothesize that bribe payments would be detectable using Benford’s Law, as bribe payments are typically based on interpersonal negotiation, and payments are typically done in cash and based on round numbers. In particular, we study whether the predictability of Benford’s Law estimates is useful for a machine learning setup in the presence of regime shifts. The 2016 Indian demonetization of some legal tender notes are informative. It allows us to compare how Benford’s Law would perform in an out-of-sample analysis whereby the legal setting of payment methods change. We document three main findings.

First, bribe payments in India violate Benford’s Law with a large deviation for payments beginning with “5”. This leading digit corresponds to the second-largest banknote of 500 rupees. We find that retail payments both online and offline satisfy Benford’s Law, consistent with findings from the previous literature, which we discuss below. However, post-2016 demonetization, which ended the usage of 1,000-rupee banknotes and introduced the 200 and 2,000 rupee note, the fit of bribe payments to Benford’s Law improved slightly.

Second, a random forest model using Benford’s Law estimates appears useful to detect bribe payments, performing slightly better than an XGBoost model based on F1 score. We also document that oversampling the data on bribes is important in improving the performance of the random forest model. However, the false-positive rate still suggests that an approach relying only on Benford’s Law estimates would not be practically feasible as analysts would still need to do manual investigations.

Third, we document that a model trained on the pre-demonetization but tested on the post-demonetization has an F1 of only 12.5%, compared to a model trained on the post-demonetization and tested on data sampled from the same period that has an F1 score of over 93.6%. Our findings highlight the importance of adjusting models to different legal settings. In particular, any follow-on research seeking to operationalize our findings should take country-specific settings into account. Where appropriate, researchers should model potential deviations from Benford’s Law in a way informed by the economic regime, such as the set of legal tender notes.

Relative to the existing research, we make two main contributions. First, we combine Benford’s Law with a machine learning algorithm and evaluate the predictive power of specific measures based on Benford’s Law. Second, we emphasize the importance of domain-specific knowledge when deploying forensic analyses, based on an economic regime shift whereby the Indian government changed the legal tender notes in India. The latter analysis shows the sensitivity of machine learning and forensic analytic methodologies in the face of changing regimes. In doing so, we highlight the importance of both feature engineering and domain knowledge in deploying these methodologies. An implication of our finding is that as payments become digitized, we expect these models to perform worse over time as bribe payments would not necessarily be clustered around legal tender combination amounts.

### 1.2 Related literature

Versions of Benford’s law has been used across various fields ranging from geophysical data analysis to studying election data. [3] studies electoral processes data from the United States and Venezuela. They use a standard tool in election forensics called “the second digit Newcomb-Benford Law” and its variations. The use of Benford’s law is also widespread in financial analyses, particularly in catching anomalies and fraud detection in financial statements, starting with [1]. [4] studies data from the Johannesburg Stock Exchange and also finds that Benford’s law is useful to classify errant financial statements, documenting that Benford’s law would detect 17 out of 17 errant statements and 3 false positives. Expanding on the Benford’s law test, [5] proposes a second-order test based on the distribution of digit frequencies of differences of ordered values in a dataset. The test appears to detect data errors and irregularities not picked up by other methods with a low false positive rate. Meanwhile, focusing on cross-border payments, [6] studies the distribution of digits in transactions between foreign and domestic businesses and find that the distribution does not conform to Benford’s law. [7] studies 27 EU member states using a dataset of transactions flagged as suspicious from reporting entities, prosecutions, and convictions of money launder. Interestingly, the study finds that the distribution of transaction values deviate less from Benford’s law when there was peer monitoring of transactions, suggesting that transaction value distributions may be informative for classifying suspicious transactions. However, the original papers did not consider machine learning tools to supplement standard tests.

Our study also builds on the research using machine learning methods for forensic analytics. For example, [8] proposes a new technique for fraud detection, which combines a Reinforcement Learning technique and a digital analysis technique. [9] applies data mining techniques to journal entries from 29 different organisations. The results from the data eventually became a valuable resource for auditors. [10] replicates these findings and extends it to additional settings. In another financial setting, [11] shows that real estate investment trust earnings data conforms to Benford’s law, suggesting that these companies either did not manage earnings, or at least that they did not manage it to the point that it could be detected. Meanwhile, [12] studies the sensitivity and specificity of a classification of potential fraud using Benford’s law compared to other heuristics. Compared to these additional forensic studies that use different tools, we focus on the most popular tool and its application with machine learning models.

To this end, our study builds upon the literature applying criteria from Benford’s law to machine learning models. Our approach to combine Benford’s Law with machine learning algorithms is most related to more recent literature combining forensic techniques with machine learning. For example, [13] uses Benford’s Law and machine learning models to detect money laundering by a company and its suppliers. Relatedly, [14] develops a method to detect credit card fraud on social media by using deep learning autoencoders in conjunction with Benford’s Law. In our research, we use random forests which are generally faster to train than autoencoders and will be more easily deployed in practice. Most recently, [15] and [16] explore the use of machine learning for fraud detection in fintech applications and money-laundering. However, [17] finds limited support for supervised machine learning methods due to the lack of high-quality training data. We contribute to this literature by showing the performance of a supervised method where we have self-reported, labeled bribery transactions in a dataset featuring both legitimate and bribe payments.

## 2. Framework and methodology

A common empirical problem involving fraud detection and forensic accounting is usually framed as classification problems, predicting a discrete class label output given a data observation. This work performs exploratory data analysis on datasets containing bribe and transactional data and aims to predict whether a given transaction is a bribe or not. The main challenge of this classification problem comes from the fact that most transactions are not fraudulent in real-world data. This results in an imbalanced dataset. Out of the many ways to deal with an imbalanced dataset, we use the oversampling technique SMOTE.

Finally, as our goal is to emphasize the importance of understanding the institutional setting with which to deploy a machine learning model, we highlight the importance of training datasets for live deployment by considering all four possible combinations of training and testing data using the pre or post demonetization data. As a baseline, we show the performance for regime-matched training and testing data in Table 1. Then, we again evaluate the performance of regime mis-matched models for training and testing data in Table 2. To compare the performance of different models, we consider a set of standard performance metrics but focus on the holistic F1 score due to the imbalance of bribe payments in the sample [18].

**Table 1:** Classification results by economic regime and machine learning model.

Panel A: Pre-Demonetization								
Model:	Random Forest				XGBoost			
	Accuracy	Precision	Recall	F1 Score	Accuracy	Precision	Recall	F1 Score
without SMOTE	95.4	53.0	3.50	6.60	95.4	54.8	3.20	6.10
with SMOTE	91.0	98.1	85.8	91.6	91.0	86.0	98.0	91.6
Panel B: Post-Demonetization								
Model:	Random Forest				XGBoost			
	Accuracy	Precision	Recall	F1 Score	Accuracy	Precision	Recall	F1 Score
without SMOTE	99.4	22.2	0.50	0.90	99.4	nan	0.0	Nan
with SMOTE	93.2	88.5	99.4	93.6	93.2	88.5	99.4	93.6

**Table 2:** The impact of regime mismatches in training and testing data.

Panel A: Trained on Pre-Demonetization and Tested on Post-Demonetization								
Model:	Random Forest				XGBoost			
	Accuracy	Precision	Recall	F1 Score	Accuracy	Precision	Recall	F1 Score
without SMOTE	94.8	7.1	49.4	12.5	96.4	4.5	26.9	7.7
with SMOTE	83.4	4.0	91.2	7.7	83.1	3.0	91.6	5.8
Panel B: Trained on Post-Demonetization and Tested on Pre-Demonetization								
Model:	Random Forest				XGBoost			
	Accuracy	Precision	Recall	F1 Score	Accuracy	Precision	Recall	F1 Score
without SMOTE	99.2	80.9	11.8	20.6	99.4	100.0	0.3	0.6
with SMOTE	87.9	5.3	89.3	10.0	87.2	3.8	88.2	7.2

The table above shows the performance of the classification using random forest and XGBoost models in the pre- and post-demonetization data, respectively. The table also shows whether the model used SMOTE or not. Panel A shows models trained up to 2016 and tested on post-2016 data. Panel B shows models trained on post-2016 and tested on data before and including 2016.

### 2.1 Background and hypotheses

Benford’s Law, also known as the Law of First Digits or the Phenomenon of Significant Digits, states that rather than following a uniform distribution, the first digits of values in a dataset should follow a different distribution: “1” should be most frequent, followed by “2”, “3”, and so on. The stability and universality of Benford’s law has made it a mainstay tool in forensic analysis to detect fraud or other irregularities, since manipulated numbers typically violate Benford’s law. [19] provides guidelines on when Benford’s Law would be applicable. Specifically, large datasets with transaction-level data, and those generated from math operations and with right-skewed values will tend to conform to Benford’s law. We apply Benford’s law to the transaction-level sample. However, before doing so, we discuss the India setting and the regime change that we use to evaluate model stability.

The Indian demonetization in November 2016 outlawed the use of the old 500- and 1,000-rupee notes. While new 500-rupee notes were reintroduced after demonetization, the 1,000 rupee notes were replaced by 2,000 rupee notes. If bribe transactions primarily take place with cash, a change in the denominations of cash will change the distribution of digits that we can expect to see. It is thus important to examine the data pre- and post-demonetization independently.

However, bribe payments violating Benford’s Law does not guarantee that it will have a practical use for detecting bribes in a sample with both legitimate and bribe payments. Therefore, we first evaluate whether bribe and non-bribe payments conform to Benford’s law, and then evaluate whether features constructed from transaction values guided by Benford’s law criteria are informative for predicting whether a transaction is a bribe payment.

### 2.2 Data source

We combine two primary data sources for our analysis. The first comes from a proprietary data set from a large retailer in India, with online and offline point-of-sales information. The second comes from a self-reported website called www.ipaidabribe.com, which was started by the United Nations and focused primarily on India. The site covers over 1,081 cities, houses over 198,000 reports, with total bribes of over 30 billion rupees as of June 2021. The second is a dataset of around one million transactions from a large Indian retailer, including point-of-sale and e-commerce transactions. All e-commerce transactions are from debit and credit cards, while point-of-sales data come from cash and card payments. Unfortunately, the dataset does not split point-of-sales data into those from cash or cards.

Our sample is from August 1, 2010, to October 31, 2019. Analyses are conducted at the transaction level, concatenating the two different datasets, where a bribe is coded as one, and a non-bribe is coded as zero. We include the day, month, and year in the dataset. We also construct additional features such as the day of the week. Since the data do not span many years, we do not use the year or month in any analyses.

Neither the bribe nor legitimate transaction data include personally identifiable information or demographic data for different parties in the transaction. Therefore, our analyses must make use of very little data, namely the transaction date and the transaction amount. Based only on these two raw data fields, we construct additional features below.

### 2.3 Techniques and models

We construct measures of the distribution of numbers across different digits as the feature engineering exercise for the machine learning application, based on intuition guided by Benford’s Law. We use these constructed features in a random forest model. Then, because bribe payments are rare (less than 4% in our data) relative to normal transactions, we train our machine learning model with synthetic minority oversampling (SMOTE) to ensure that model node splitting’s are informative for the portion of data of interest: those similar to bribe payments. We discuss the variable constructions and machine learning process below.

Using imbalanced data in a classifier will bias it towards the majority class. To counteract this imbalance, a data augmentation technique called the Synthetic Minority Oversampling Technique (SMOTE) can be used [20]. This technique reduces data imbalance by oversampling the minority class using a nearest neighbors’ approach. For example, if one uses five nearest neighbors and the amount of over-sampling is 300%, three of the five neighbors are chosen for a synthetic sample. Synthetic samples are generated by taking a convex combination across all features, with weights drawn from a standard uniform random variable, between the actual sample and its chosen nearest neighbor. In our analyses, we use a standard of five nearest neighbors.

The machine learning models are a random forest model from the *scikit-learn* library and XGBoost from the *XGBoost* library in Python. The former builds multiple decision trees and merges them to get a more accurate and stable prediction. Many relatively uncorrelated decision trees are trained to minimize the out-of-bag error. We tune hyperparameters using validation curves as a guide to arrive at a suitable combination, keeping several parameters at their default values for simplicity. The latter (XGBoost) stands for “Extreme Gradient Boosting.” Like to the random forest, XGBoost is a decision-tree-based machine learning algorithm. But unlike the random forest, XGBoost uses a gradient descent approach to build one tree at a time rather than independently. The methodology to arrive at convergence is slightly different from the random forest, which relies on a Gini impurity metric. Instead, XGBoost minimizes a regularized (L1 and L2) objective function.

For all our analyses, we consider a dataset split into 75% of randomly drawn observations into the training set and the remaining 25% into the testing dataset. When the sample come from mismatched economic regimes, we sample the number of data points such that the overall sample at 75% from one economic regime and 25% from another economic regime.

## 3. Does Benford’s law apply?

**Fig 1:**
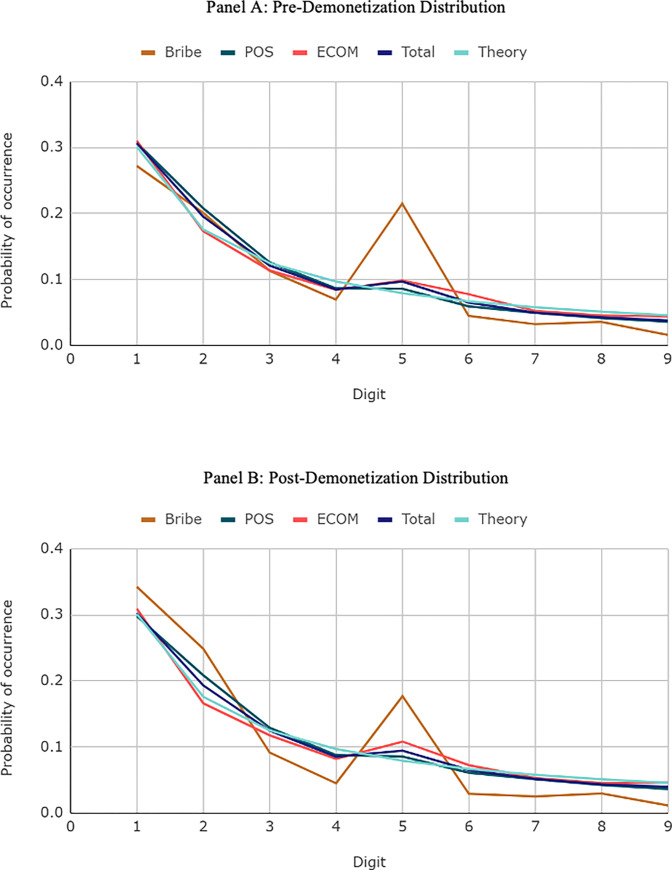
Distribution of first digits of transactions by type and economic regime. **A.** Pre-Demonetization Distribution. **B.** Post- Demonetization Distribution.

Fig 1 below visualizes the distribution of the first digit values. Panel A shows the distribution pre-demonetization, and Panel B shows the distribution post-demonetization. In both panels, we find gthat bribe payments exhibit the largest deviation from Benford’s Law predictions compared to normal transactions. In addition, both pre- and post-demonetization bribe samples show deviation around 5. Benford’s law predicts an occurance of 0.079 for the first digit value 5. Pre demonetization it is 0.215 while post-demonetization it is 0.177. This leading digit corresponds to the second-largest banknote of 500 rupees.

However, there are some other subtle differences between the samples. Post-demonetization, we find a significant deviation around the digit 2 post demonetization when 200- and 2,000-rupee notes were introduced. These descriptive statistics show that the demonetization of 1,000-rupee bills and the introduction of 200- and 2,000-rupee bills changed the distribution of the first digit of bribe payment amounts.

In addition, the weights for digits beginning with 3, 4, 6, 7, 8, and 9 are below what Benford’s law predicts. Interestingly, we see similar but less extreme deviations both in the point-of-sales purchase amounts and the e-commerce amounts. The “total” line aggregates point-of-sales, e-commerce, and bribe payments together. We see that all together, the pattern fits most closely with Benford’s Law. These empirical patterns suggest that Benford’s Law has the potential to be used to detect bribe payments from other kinds of normal transactions.

Although the distributional changes for the first digit show stark patterns, the use case for the first digit distribution for predicting transaction-by-transaction classifications is not obvious. To operationalize this distribution, we consider not just the first digit but also the distribution of other digits as well. Importantly, we adopt a machine learning approach instead of a standard linear model approach as the non-linearities around the distribution of digits based on different units (1’s, 10’s, 100’s, 1,000’s) may feature in non-trivial ways that are informative for prediction. Therefore, although visually the distributions of first digits may look similar for the pre- and post-demonetization samples, the information content contained in the distribution of first digits depends on the economic regime. When different units’ leading digits are used in the machine learning model, as a non-linear combination of data points, we are unable to ex ante evaluate whether the models would be highly sensitive to these subtle differences in the data, since we do not have a strong prior as to how different digits should combine to inform us about whether a payment is a bribe payment or not.

## 4. Classification results

### 4.1 Baseline results

The S1 Appendix shows summary statistics, and Table 1 below shows four standard measures of performance: accuracy, precision, recall, and F1 score. Panel A shows the results for the analyses using pre-demonetization data, and Panel B shows the results of the analyses using post-demonetization data. As discussed in the methodology section, we train the model on a randomly selected 75% of the data, and the training dataset is the remainder, split by pre- and post-demonetization separately. In other words, the pre-demonetization results in Panel A are both trained and tested on pre-demonetization results. We explore what happens when when a model trained on pre-demonetization data is tested on post-demonetization data in Section 4.2.

Table 1 shows that although the accuracy is roughly the same when using SMOTE and not using SMOTE, there is a dramatic improvement when using SMOTE compared to using the full highly unbalanced data. The high accuracy is due to the highly imbalanced nature of the data, as a simple prediction that all would have high true negative rates. Given that most transactions are legitimate transactions and not bribes, the accuracy is high. A composite measure like the F1 score also shows a dramatic increase due to improved precision and recall of the models using SMOTE.

The table below shows the performance of the classification using random forest and XGBoost models in the pre- and post-demonetization data, respectively. The table also shows whether the model used SMOTE or not. Panel A shows models trained and tested on pre-demonetization data and Panel B shows models trained and tested on post-demonetization data. The time taken to train the random forest models are between 1 to 3 minutes for the random forest models without and with SMOTE, and the XGBoost models take between 0.5 to 1.5 minutes to train. Unsurprisingly, the XGBoost algorithm is much faster to train. Therefore, all the models that we consider can be trained and deployed in a practice with little time and computation costs.

**Fig 2:**
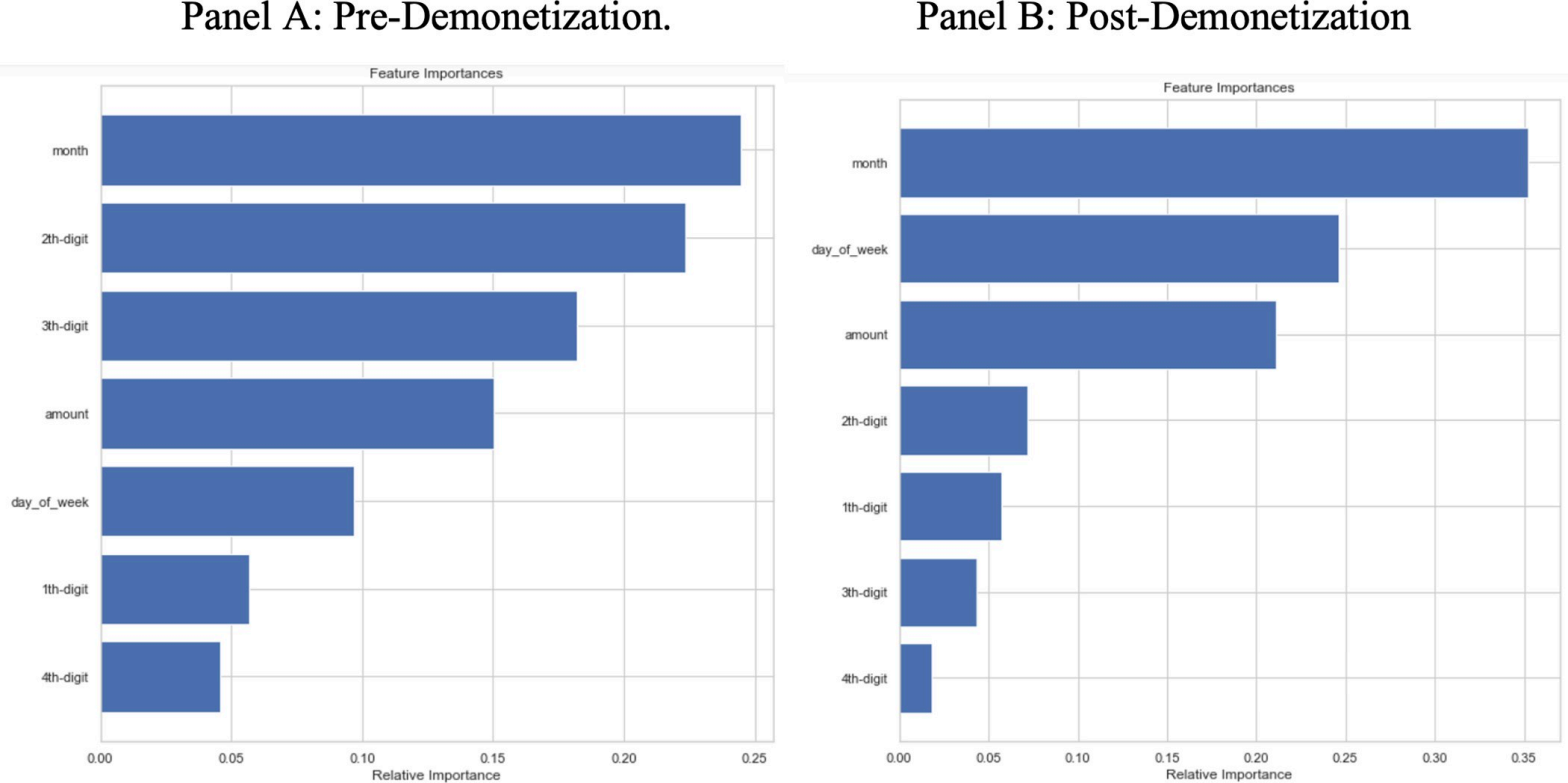
Variable importance plots by economic regime for random forest model. **A.** Pre-Demonetization. **B.** Post-Demonetization.

Fig 2 shows the variable importance plot for the random forest models trained and tested on pre- and post-demonetization data respectively. Both panels show the variable importance plot of the random forest models from the pre- and post-demonetization data. To allow the model to be usable in practice, we do not include the date as a variable for prediction, although we extract the day of the week and month for use in the model. However, we include the day of the week as a potential explanatory variable. Pre-demonetization, we find that the calendar month is the most important variable, followed by the value of the second digit, third digit, and total amount of the payment. In the post-demonetization period, the calendar month remains the most important predictor, followed by the day of the week, amount, and the second digit. Interestingly, although Benford’s Law evaluation focuses on the distribution of the first digits, we find that the more informative transaction variable is the second digit. The first digit ranks in the bottom three out of the eight variables are considered in both the pre- and post-demonetization models.

**Fig 3:**
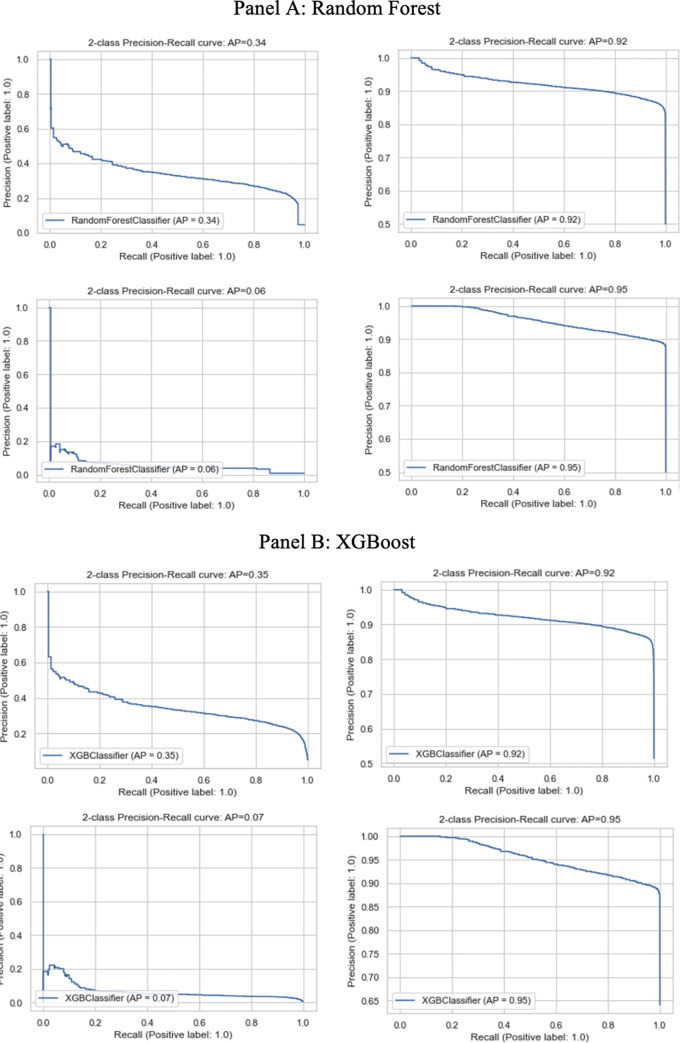
Precision-Recall curve by model and train-test regimes. The subplots on the left show the model without SMOTE and those on the right use SMOTE to oversample the bribe payments. The subplots on the top train and test the model on pre-demonetization data and those on the bottom train and test the model on post-demonetization data. Panel A shows results for the random forest model and Panel B shows the results for the XGBoost model.

The precision-recall curve summarizes the trade-off between the true positive rate and the positive predictive value for a predictive model using different probability thresholds. Both are useful in cases where there is an imbalance in the observations between the two classes. A no-skill classifier cannot discriminate between the classes and would predict a random or constant class in all cases. The no-skill line changes based on the distribution of the positive to negative classes. It is a horizontal line with the value equal to the ratio of positive cases in the dataset. For a balanced dataset, this value is 0.5. The two right subplots in Fig 3 show that both the random forest and XGBoost model with SMOTE performs much better than that without SMOTE (left subplot).

**Fig 4:**
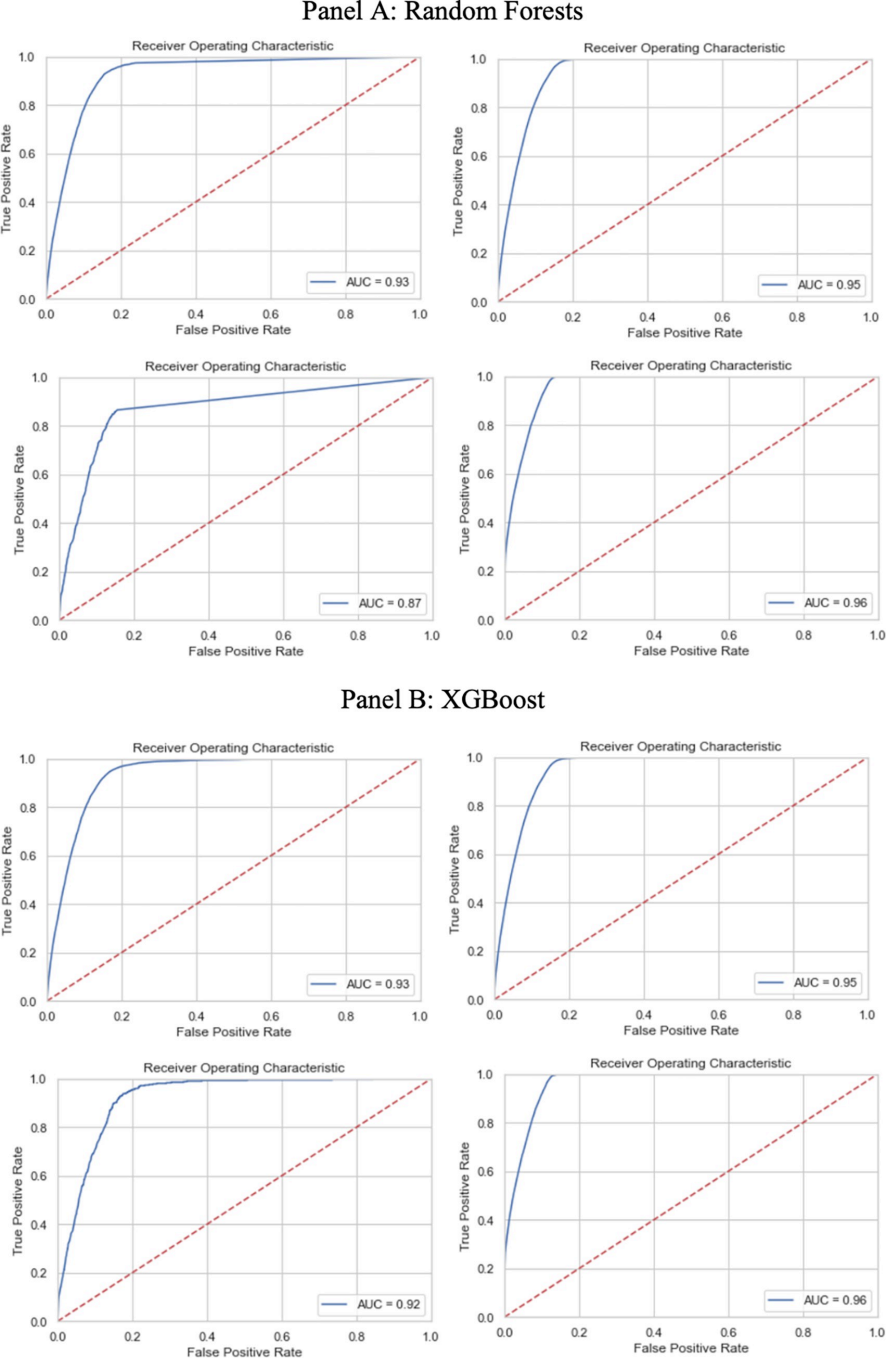
Receiver operating characteristic curves by model and train-test regimes. The subplots on the left show the model without SMOTE and those on the right use SMOTE to oversample the bribe payments. The subplots on the top train and test the model on pre-demonetization data and those on the bottom train and test the model on post-demonetization data. Panel A shows results for the random forest model and Panel B shows the results for the XGBoost model.

The receiver operating characteristics (ROC) curves trade-off between the true positive rate and false positive rate for a predictive model using different probability thresholds. When the AUC for the ROC curve is 0.5, it means that the classifier cannot distinguish between Positive and Negative class points. This implies that the classifier predicts a coin-flip random class for all the data points. When the area under the curve is 1, the classifier can perfectly distinguish between all the Positive and the Negative class points. The two right subplots in Fig 4 show that the ROC of both the random forest and XGBoost model with SMOTE performs much better than that without SMOTE (left subplots). The plots also suggest that the eight variables we considered perform better in the post-demonetization than the pre-demonetization data, although Table 1 suggests that the difference in F1 is not large.

Overall, contrary to the hypothesis that Benford’s Law-type measures would not be informative for detecting bribe payments, we find evidence more consistent with the alternative hypothesis, that one can indeed use the distribution of digits to detect bribe payments in a highly imbalanced dataset.

### 4.2 Importance of regime-specific models

In this next section, we study whether the models’ performance reflects the information in bribe payments that are not legitimate transactions and that depend on the economic regime. In particular, the regime that we evaluate is the different sets of legal tender notes. Therefore, we compare the performance of models trained in one regime but tested in another. To show our results’ robustness and emphasize the importance of economic regime alignment under which the models are trained and tested, we consider both cross-regime specifications. If we use the pre-demonetization data as the training set and the post-demonetization data as the testing set, we obtain the following results in Panel A of Table 2. To further highlight the regime dependency of the model, Panel B is the converse of Panel A: using the post-demonetization data as the training set and the pre-demonetization data as the testing set, we obtain the following results.

Therefore, we compare the performance of models trained in one regime but tested in another. We consider both cross-regime specifications to show our results’ robustness and emphasize the importance of economic regime alignment under which the models are trained and tested. If we use the pre-demonetization data as the training set and the post-demonetization data as the testing set, we obtain the following results in Panel A of Table 2. To further highlight the regime dependency of the model, Panel B is the converse of Panel A: using the post-demonetization data as the training set and the pre-demonetization data as the testing set, we obtain the following results.

## 5. Conclusion

This paper shows that forensic accounting techniques can be used with machine learning methodologies to detect bribe payments in a combined sample of bribes and normal transactions. In particular, given the extreme imbalance in the data, oversampling the bribe payments in training the model is important and improves recall by over ten-fold. However, bribe payments appear regime-specific and depend on the combination of legal tender notes in an economy. Using a model trained before the Indian demonetization in 2016 and used after the demonetization sees a large decrease in performance between 10 to 30% relative to models trained and tested in the same regime.

Our research shows the importance of domain or regime-specific knowledge in using these procedures. Users should not simply start a model and let it run always. Instead, analysts using such tools should consider the stability of their results in different settings, and if necessary, consider re-training the model on a new dataset if the underlying economic framework justifies it. Our results is a first step towards undersatnding how the underlying institutional setting in a payment system affects forensic analytic performance. Further research can take our existing results as a starting point for developing a potential diagnostic tool to detect shifts in the underlying data which may inform users whether a model is likely to be stale and needing re-calibration. In addition, we only consider the use of Benford’s Law in detecting bribe payments. Follow-on work may also consider alternative forensic analyses tools and distributions.

## Supporting information

S1 Appendix (DOCX)

S1 File (ZIP)
