# Supplementary material for: Using forensic analytics and machine learning to detect bribe payments in regime-switching environments: Evidence from the India demonetization
Source: PLoS One. 2022 Jun 9;17(6):e0268965. doi: 10.1371/journal.pone.0268965 (PMC9182657; doi:10.1371/journal.pone.0268965)
Supplement: S1 Appendix — (DOCX) [file pone.0268965.s001.docx]

# Appendix: Summary Statistics

In this section, we discuss more details on the summary statistics of the bribe payments pertaining to day-of-week and month-of-year seasonality.

In terms of day-of-week seasonality, we find that bribes take place most on Mondays, then followed by Fridays. This may be because offices open on Monday after staying shut for one or two consecutive days. The maximum number of non-bribe transactions take place on Saturdays and Sundays. This is because the majority of shopping is done on the weekends.

**S1 Table.** Distribution of bribes vs non-bribe payments across days-of-week

| **Day of Week** | **Distribution of Bribes** | **Distribution of Non-Bribes** |
| --- | --- | --- |
| Monday | 18.55% | 13.14% |
| Tuesday | 12.07% | 12.89% |
| Wednesday | 12.41% | 13.21% |
| Thursday | 15.09% | 13.21% |
| Friday | 16.50% | 13.82% |
| Saturday | 14.00% | 17.43% |
| Sunday | 11.38% | 16.30% |

In terms of monthly seasonality, the maximum number of bribes are observed in the month of October which may be due to the occurrence of the two major Indian festivals of Dussehra and Diwali in October. The non-bribe data are uniformly distributed across the months.

**S2 Table.** Distribution of bribes vs non-bribe payments across months

| **Month** | **Distribution of Bribes** | **Distribution of Non-Bribes** |
| --- | --- | --- |
| January | 3.08% | 8.29% |
| February | 3.28% | 7.33% |
| March | 13.11% | 8.08% |
| April | 7.44% | 8.18% |
| May | 5.83% | 8.46% |
| June | 8.15% | 8.10% |
| July | 5.92% | 8.35% |
| August | 11.45% | 8.27% |
| September | 8.72% | 8.02% |
| October | 22.15% | 8.74% |
| November | 6.31% | 8.67% |
| December | 4.56% | 9.51% |
